# Fiber-Templated 3D Calcium-Phosphate Scaffolds for Biomedical Applications: The Role of the Thermal Treatment Ambient on Physico-Chemical Properties

**DOI:** 10.3390/ma14092198

**Published:** 2021-04-25

**Authors:** Aura-Cătălina Mocanu, Florin Miculescu, George E. Stan, Andreea-Mădălina Pandele, Mihai Alin Pop, Robert Cătălin Ciocoiu, Ștefan Ioan Voicu, Lucian-Toma Ciocan

**Affiliations:** 1 Department of Metallic Materials Science, Physical Metallurgy, University Politehnica of Bucharest, 313 Splaiul Independentei, J Building, RO-060042 Bucharest, Romania; mcn_aura@hotmail.com (A.-C.M.); ciocoiurobert@gmail.com (R.C.C.); 2 National Institute of Materials Physics, 405A Atomistilor Street, RO-077125 Măgurele, Romania; george_stan@infim.ro; 3 Department of Analytical Chemistry and Environmental Engineering, University Politehnica of Bucharest, 1-7 Gh. Polizu, RO-011061 Bucharest, Romania; pandele.m.a@gmail.com (A.-M.P.); svoicu@gmail.com (Ş.I.V.); 4 Advanced Polymer Materials Group, University Politehnica of Bucharest, 1-7 Gh. Polizu, RO-011061 Bucharest, Romania; 5 Department of Materials Science, Faculty of Materials Science and Engineering, ICDT, University Transilvania of Brasov, 10 Institutului, RO-500484 Brasov, Romania; mihai.pop@unitbv.ro; 6 Prosthetics Technology and Dental Materials Department, “Carol Davila” University of Medicine and Pharmacy, 37 Dionisie Lupu Street, RO-020022 Bucharest, Romania; tciocan@yahoo.com

**Keywords:** marble, graphene, *Luffa*, sintering ambient, reinforced bio-products, biomedical, applications

## Abstract

A successful bone-graft-controlled healing entails the development of novel products with tunable compositional and architectural features and mechanical performances and is, thereby, able to accommodate fast bone in-growth and remodeling. To this effect, graphene nanoplatelets and *Luffa*-fibers were chosen as mechanical reinforcement phase and sacrificial template, respectively, and incorporated into a hydroxyapatite and brushite matrix derived by marble conversion with the help of a reproducible technology. The bio-products, framed by a one-stage-addition polymer-free fabrication route, were thoroughly physico-chemically investigated (by XRD, FTIR spectroscopy, SEM, and nano-computed tomography analysis, as well as surface energy measurements and mechanical performance assessments) after sintering in air or nitrogen ambient. The experiments exposed that the coupling of a nitrogen ambient with the graphene admixing triggers, in both compact and porous samples, important structural (i.e., decomposition of β-Ca_3_(PO_4_)_2_ into α-Ca_3_(PO_4_)_2_ and α-Ca_2_P_2_O_7_) and morphological modifications. Certain restrictions and benefits were outlined with respect to the spatial porosity and global mechanical features of the derived bone scaffolds. Specifically, in nitrogen ambient, the graphene amount should be set to a maximum 0.25 wt.% in the case of compact products, while for the porous ones, significantly augmented compressive strengths were revealed at all graphene amounts. The sintering ambient or the graphene addition did not interfere with the *Luffa* ability to generate 3D-channels-arrays at high temperatures. It can be concluded that both *Luffa* and graphene agents act as adjuvants under nitrogen ambient, and that their incorporation-ratio can be modulated to favorably fit certain foreseeable biomedical applications.

## 1. Introduction

Incipient discoveries in the realm of ceramic materials for bone healing applications can be dated back to over half a century ago [1,2]. However, it was not until the beginning of the 1980s that the medical community started using and marketing calcium phosphates (mostly hydroxyapatite, HA, Ca_10_(PO_4_)_6_(OH)_2_) for restorative dental procedures and bone defect repair [1,3]. The ongoing need for advanced bone surgical procedures and bone-mimicking structures is directly correlated with the increase of the elderly population and, thereby, with the frequency upsurge of cases when bone is unable to repair itself (i.e., associated pathological conditions, large defects caused by severe traumas or tumor resections) [4,5,6,7].

The well-known drawbacks of the standard biological approaches (e.g., autografts and allografts) [4,8,9,10,11] have put a concerted focus on alternative solutions based on calcium phosphates (CaP)-based products (i.e., HA, β- and α-tricalcium phosphate (TCP, Ca_3_(PO_4_)_2_), brushite (DCPD, CaHPO_4_·2H_2_O), calcium pyrophosphate (CPP, Ca_2_P_2_O_7_), or the combination of them) [12,13,14,15,16,17,18]. The extension of the range of biomedical applicability requires continuous improvements of this category of bioactive materials. Of upmost importance for synthetic bone graft substitutes is their capacity to adapt to the implantation sites and aid the reparatory osteogenesis process without generating dramatic responses (i.e., immunologic, allergenic, mutagenic effects) [9,12,19,20].

Another widely-discussed requirement is the dissolution and resorption degree of the bone graft substitute materials [5,13,21] and the favorable hydroxyapatite formation [22,23,24]. Therefore, HA with/without β-TCP/α-TCP is usually destined for the fabrication of 3D compact and porous structures, while DCPD can be used alone or in combination with any other calcium phosphate for the preparation of bone cements and bone fillers [13,16,25,26]. Products fabricated predominantly of β-TCP and α-TCP are rarely suggested for load-bearing applications owing to their low resistance and high resorption rates and, thus, insufficient time for a proper bonding with the surrounding bone tissue [13,19,27]. However, the addition of different ratios of TCP materials was proposed for a controlled degradation rate and an improved protein adsorption on the implant surface [5,28,29]. Supplementary CPPs were also found promising for the development of bone grafting [16,17,30].

However, for an optimal bone regeneration, the CaP-based materials should further fulfil specific bone-mimicry prerequisites, which encompass both architectural and mechanical features. Ideally, the replication of the interconnected porous and vascular-branched structure of the bone is targeted for such products so as to further promote the remodeling process, culminating with an adequate new bone formation [12,27,31].

The concerning economic aspects circumscribed to waste disposal recently engendered strategies oriented towards reviving some of the natural materials/resources (i.e., marine shells and bones, marble, plant and animal fibers, wood chips) by sustainably recycling them into value-added parts for different industries (bio-medical included) [4,8,32,33,34,35,36,37,38]. The use of vegetable fibers (sourced in considerable amounts and at low costs) was tackled, mostly for the mechanical and thermal reinforcement of composites, after being chemically processed or polymer-bonded to ceramic matrices [36,37]. Rarely were the *Luffa* fibers employed as sacrificial templates for the fabrication of hierarchically porous structures, in the absence of binding additives [4,10,39]. When exposed to high temperatures, *Luffa* fibers are prone to decomposition by combustion or carbonization (depending on the parameters and ambient of the thermal treatment) [4,36,37,40]. This can generate favorable and complex networks of pores and tortuous channels, stemming from the interior towards the exterior of the ceramic bodies [4,39].

The bone-defect-shaped product should also withstand significant static (e.g., hardness, compressive strength) and dynamic (e.g., fatigue and crack propagation resistance) mechanical stresses [8,31]. Consequently, CaPs have long been investigated to reduce their susceptibility to implant loosening and failure (mainly owned to the intrinsic brittleness, low toughness, ductility, and wear resistance) and improve their mechanical reliability [5,9,25,41]. Mechanically reliable products can be achieved by merging a reinforcement agent into the ceramic matrix [42]. The beneficial effects generated by the addition of graphene materials as a toughening and reinforcing phase have been ascribed to their unique physical (i.e., thermal stability, high electrical conductivity, and mechanical strength) and biological (i.e., bioactivity, antibacterial effect) properties [41,43,44,45]. However, the graphene-CaP composites were suggested only a few years ago [9,46]. An evolution from laborious chemical preparation routes of such composites towards time and cost efficient and completely reproducible technologies can be lately acknowledged [41,47]. A recent preliminary study exposed the successful homogenous incorporation of graphene nanoplatelets directly into the ceramic matrix, which helped mend the mechanical pitfalls and improve the biofunctionality of both the compact and porous developed products [10].

Adequate spatial porosity and mechanical resistance properties can be attained by sintering processing in various ambient environments via diffusion, evaporation, and condensation mechanisms [28,29,48]. Several studies outlined the typical behavior and phase transition of CaPs (i.e., HA, DCPD) with/without graphene materials, in oxidative, reductive, or inert ambient environments [2,27,44,45,49,50,51,52,53]. However, the benefits of the sintering ambient on the overall features of HA and DCPD-composite assemblies with *Luffa* fibers remains, to the best of our knowledge, an untouched area of research.

Based on the above-mentioned criteria and the absence of similar dedicated studies, this research attempts to demonstrate the critical influence of the sintering ambient (air vs. nitrogen) for compositional optimization and performance modulation of the 3D bio-products destined for non- and load-bearing orthopedic applications. The tailoring of bone graft substitutes that may impact the biological response starts with the fabrication of the ceramic matrix by a reproducible, facile, and cost-efficient conversion of dolomitic marble (autochthonous natural resource), as previously reported in Refs. [26,34,35]. It is important to mention their positive in vitro response, already investigated throughout various extensive studies [26,34,54]. The composite assemblies will further rely on the homogenous incorporation of graphene nanoplatelets as mechanical reinforcement agent, as recently presented in Ref. [10]. The creation of the hierarchical porous configuration/permeation will be induced by the *Luffa* fibers’ ability to act as sacrificial template, regardless of the admixed graphene amount [4,39].

In this study, the influence of a key factor—the sintering ambient (air vs. nitrogen)—on the successful join of all scaffold component elements (ceramic matrix, mechanical reinforcing additive, and porogen agent) is reported nowhere in the literature and will be evaluated for the first time. The proposed study is the logical continuation of our previous reported research, which aimed to demonstrate the biologically-safe use of the *Luffa* fibers and their ability to generate internal porous arrays [4], and to preliminarily investigate the possibility of combining the two agents, one as sacrificial porogen template and another as mechanical reinforcement [10]. Several valuable particularities and defining aspects for the 3D bio-products (compact and porous) will be further exposed within the current study: (i) compositional variability of the ceramic matrix; (ii) contrasting mechanical behaviors; and (iii) particular morphological features extended to the porous internal architectures, function of the sintering ambient (oxidative vs. reductive), and the concomitant addition of graphene nanoplatelets. The optimum graphene phase amount and sintering ambient will be finally delineated for the appropriate scaffold processing in close connection with the bone regeneration requirements.

## 2. Materials and Methods

### 2.1. Sample Preparation

The raw natural fibers of *Luffa cylindrica* (Lu) were purchased from local suppliers and were further used for the development of 3D products without any chemical or physical treatment.

The fabrication of the 3D bio-products involved a 3-step process: (i) *the synthesis of the calcium phosphate-based matrix* (*CaP*) was achieved by the conversion of raw marble into calcium hydroxide and its further reaction with phosphoric acid (H_3_PO_4_, 85%, Sigma-Aldrich, St. Louis, MO, USA) and subsequent thermal processing by employing a facile, cost-efficient, and reproducible route, as reported elsewhere [26,34,54]; (ii) *the reinforcement of the ceramic matrix* was performed by incorporation of graphene nanoplatelets (Gr), grade M (XG Sciences, Lansing, MI, USA) with thickness and diameter of 6–8 nm and 5 μm, respectively, by mechanical mixing (Inversina-2L-manual, Bioengineering AG, Wald, Switzerland) and ultrasonic dispersion (SONICS Vibra Cell, Sonics and Materials, Inc., Newtown, CT, USA) for 30 min each; the Gr amounts were chosen as 0.00, 0.25, 0.50, and 1.00 wt.%. At this stage, compact pellets (CP) were obtained from half the amount of the prepared mixtures by isostatic pressing in cylindrical molds (Φ 10 mm) at 2.5 MPa (WK 50 FH PRO, Bernardo, Linz, Austria), resulting in one sample set of CP for each Gr amount. The remaining CaP and Gr mixture from each batch was further blended with Lu fibers at a constant mass ratio of 14 wt.% of the reinforced ceramic matrix; four sample sets of porous pellets (PP) were subsequently framed by the same compressing process as the compact ones; (iii) *the thermal treatment* of the CP and PP samples was performed in two ambient environments–air (at 1200 °C for 8 h in electric furnace, Nabertherm GmbH, Liliethal/Bremen, Germany) and nitrogen (at 1200 °C for 8 h in electric furnace, Nabertherm GmbH, Liliethal/Bremen, Germany). The cooling was attained overnight in the furnace. Afterwards, the PP samples were washed under a distilled water flow and dried in the autoclave (100 °C for 2 h) [4]. Finally, both CP and PP samples were stored in sterile Petri dishes. The experimental procedure for the ceramic mixture preparation and 3D product processing, prior and post sintering in both ambients (air and nitrogen), is presented in Figure S1 (Supplementary Materials).

Plan-parallel surfaces were obtained by grinding both CP and PP samples on abrasive SiC papers (grits P600–2500). The fractographic surfaces were achieved by subjecting some of the obtained samples, from each type and for each Gr amount, to a three-point bending test.

### 2.2. Physico-Chemical Characterization

#### 2.2.1. X-ray Diffraction Investigation

The structure and phase composition of the samples was inferred by X-ray diffraction (XRD) in symmetric (θ–θ) geometry with the help of a Bruker D8 Advance diffractometer (Bruker AXS Advanced X-ray Solutions GmbH, Karlsruhe, Germany) equipped with a copper X-ray tube (λ = 1.5418 Å) and a high efficiency LynxEye™ linear detector. The patterns were acquired in the 2θ range 15–55° with a step size of 0.02° and a dwell time of 1 s/step. Each sample was rotated during measurement with a speed of 30 rotations/minute, so as to average over compositional in-homogeneities (if any).

#### 2.2.2. FTIR Spectroscopy Analysis

Complementary to XRD, the specimens were examined by Fourier-transform infrared (FTIR) spectroscopy in attenuated total reflectance (ATR) mode. The FTIR-ATR spectra were recorded in the wave number range of 500–1200 cm^−1^ at a resolution of 4 cm^−1^ by employing a PerkinElmer BX Spectrum II apparatus (PerkinElmer Corporation, Waltham, MA, USA) equipped with a Pike MIRacle (PIKE Technologies, Madison, WI, USA) ATR attachment having a diamond–zinc selenide crystal (with a diameter of 1.8 mm).

#### 2.2.3. Morpho-Compositional SEM/EDS Evaluation

The morpho-compositional analysis of the products thermally treated in air or nitrogen ambient was performed by scanning electron microscopy (SEM) with a Philips XL 30 ESEM TMP microscope (Hillsboro, OR, USA) coupled with an EDAX Sapphire spectrometer for the energy X-ray dispersive spectroscopy (EDS) evaluation. The samples were investigated at an acceleration voltage of 25 kV and working distance of 10 mm over five randomly chosen areas.

#### 2.2.4. Dimensional Shrinkage and Mass Loss Determination

Dimensional shrinkage was determined by measuring the compact and porous 3D products (diameter and height) prior and after sintering in air or nitrogen ambient. The corresponding total mass loss was determined by weighing the samples on a calibrated four decimal analytical balance (Kern & Sohn GmbH, Balingen, Germany). The results will be presented as arithmetic average ± standard deviation (*n* = 5; five sets of measurements). The detailed numerical data is expressed in Tables S1 and S2.

#### 2.2.5. Nano-Computed Tomography Reconstruction Analysis

The nano-CT analysis was conducted on Skyscanner 2211 X-ray tomograph (NanoCT, Bruker, Kontich, Belgium) without using filters. The analysis parameters were set as follows: voltage of 140 kV, current of 200 µA, and data acquisition with a rotation set of 0.2° and seven average frames per capture. The images were reconstructed with the NRecon 1.7.1.6 software from Bruker microCT with the following reconstruction setting: post-alignment 3, smoothing 4, beam hardening correction 22%, and ring artefact correction 13. The porosity of the samples was determined with the 3D analysis software from Bruker microCT, which implied the following tasks: thresholding, despeckling, and 3D analysis, with no supplementary image processing.

#### 2.2.6. Contact Angle Measurements

The wettability degree was evaluated by contact angle (CA) measurements with two different polar and dispersive wetting agents: water (W) and ethylene glycol (EG), using a Krüss Drop Shape Analyser—DSA100 (A. Krüss Optronic GmbH, Hamburg, Germany). The experiments were performed at constant parameters: temperature of 25 ± 1 °C and room humidity of 45 ± 5%. The results were captured at 1 s after droplet deposition on the CP sample surface. Given the fast impregnation encountered for the PP samples, no conclusive CA images could be captured. Therefore, the surface free energy (SFE) was determined only for the CP samples by the Owens, Wendt, Rabel, and Kaelble (OWRK) method [55,56]. The results will be presented as arithmetic average ± standard deviation (*n* = 5).

#### 2.2.7. Mechanical Properties Evaluation

The compression strength of CP and PP samples, for each Gr amount, was tested with Walter + Bai AG, Loehningen (LFV300) Testing Machine equipment (Schaffhausen, Switzerland). The employed test speed was set at 1 mm/min and the acquisition rate at 0.01 s. The measurements were performed on five samples from each type and at each Gr amount. The results are presented as arithmetic average ± standard deviation (*n* = 5).

## 3. Results and Discussion

### 3.1. XRD Investigation

**Figure 1:**
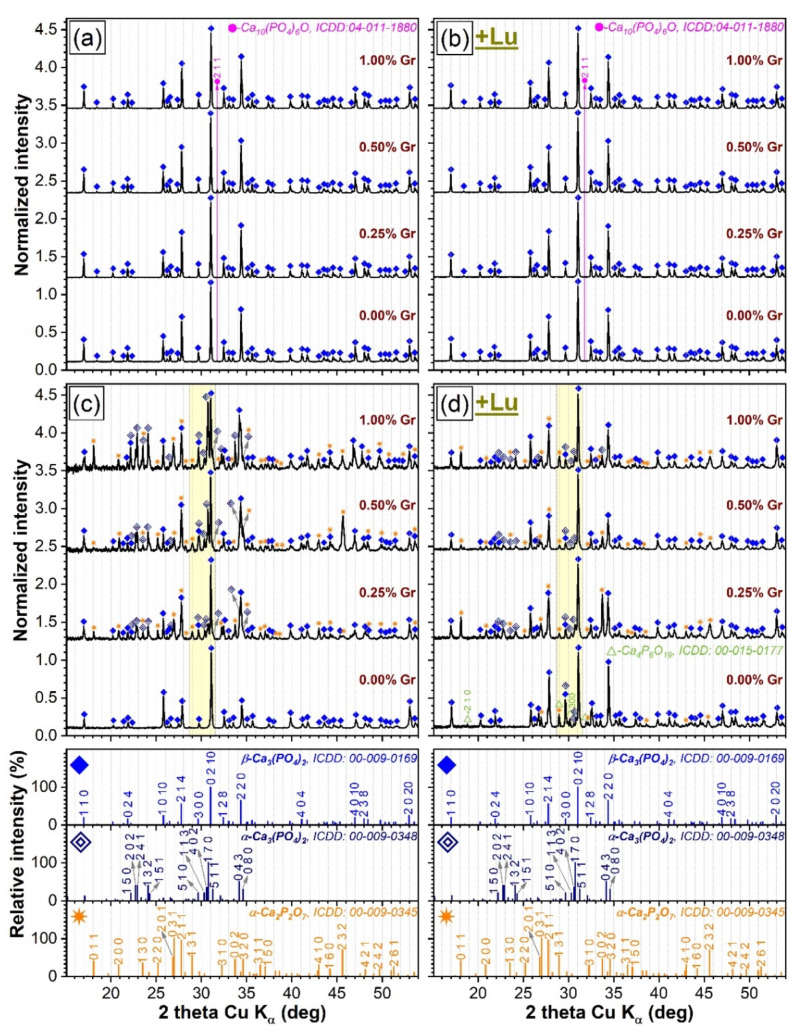
The comparative XRD patterns of the (**a**,**c**) compact and (**b**,**d**) porous samples thermally-treated in (**a**,**b**) air and (**c**,**d**) nitrogen ambient at 1200 °C/8 h. On the bottom of each graph column are presented the ICDD reference diffraction files of the main constitutive crystalline phases (i.e., β-TCP, α-TCP, and α-Ca_2_P_2_O_7_).

**Figure 2:**
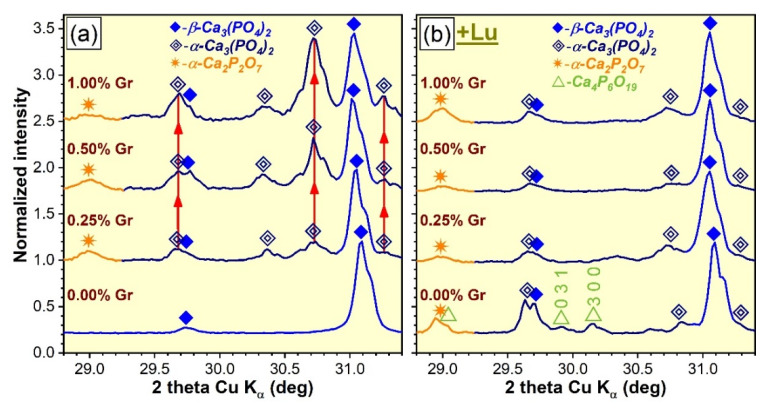
The XRD diagrams of the (**a**) compact and (**b**) porous specimens thermally-treated in nitrogen ambient at 1200 °C/8 h, zoomed in the angular region 2 θ = 28.8–31.4° (highlighted in yellow in Figure 1) to emphasize the β-TCP phase transformation.

The XRD patterns of the CP and PP specimens, fabricated by thermal processing at 1200 °C/8 h air or nitrogen ambient, are presented comparatively in Figure 1. Important differences were observed depending on the nature of the heat-treatment atmosphere. If all (compact and porous) samples thermally treated in air largely contained a β-TCP phase (ICDD: 00-009-0169) and oxyapatite [Ca_10_(PO_4_)_6_O, ICDD: 04-011-1880] as a residual minor component (Figure 1a,b), (both CP and PP) samples fabricated under a reductive nitrogen ambient consisted of β-TCP (as the major phase) and α-TCP (ICDD: 00-009-0348) and α-Ca_2_P_2_O_7_ (ICDD: 00-009-0345) (as supplemental phases). One exception was evidenced: the compact sample without Gr addition (Figure 1c), which was comprised of β-TCP only, without any residual phases, at the detection limit of the employed apparatus. Another particularity was that the corresponding porous specimen (without Gr) entailed minor contents of a catena-hexaphosphate like-phase (Ca_4_P_6_O_19_, ICDD: 00-015-0177) (Figure 1d). Interestingly, in the case of CP samples synthesized under nitrogen environment, a gradual decomposition of the β-TCP into α-TCP with the Gr additive increase (Figure 2a) was observed, with the α-Ca_2_P_2_O_7_ phase (emerging when Gr was added), but overall major changes were not recorded. The corresponding porous specimens registered a dissimilar behavior, with both α-TCP and α-Ca_2_P_2_O_7_ phases being formed as well (Figure 2b) but, with their evolution, experiencing no obvious development trend.

If the diffraction maxima of the β-TCP and α-TCP phases elicited relative intensities similar to those of the lines of their corresponding ICDD reference files (shown for comparison at the bottom of Figure 1), in the case of α-Ca_2_P_2_O_7_-like phase, a series of modifications were noticed (please see the comparative evolution of the solitary 011 and 232 diffraction lines, positioned at 2θ ≈ 18.1° and 2θ ≈ 45.6°, not superimposed by the dominant diffraction peaks of the β-TCP phase). The modification of the relative intensities of the diffraction lines indicates that deviations from the atomic site positions in the unit cell with respect from the reference/normal ones occur. Further systematic insightful studies are envisaged in the near future to unveil the underlying causes.

Worth mentioning is the fact that no additional structural interferences were recorded as a result of the interaction between the ceramic matrix and the generated Lu char in either sintering ambient [4]. Moreover, the absence of Gr/graphene oxide and the ligno-cellulosic phases linked to the presence of the natural fibers [4,37] unveiled the complete reduction/elimination of the two agents during sintering, regardless of the chosen ambient.

The structural changes highlighted for the CP and PP samples sintered in nitrogen can be attributed to the (i) biphasic HA/DCPD composition of the source ceramic powder [26,34], (ii) type of sintering ambient, and (iii) Gr admixed amounts.

(i). The DCPD phase, enclosed in HA/DCPD mixtures, was occasionally found to partially or fully transform, by dehydration and condensation, into γ-Ca_2_P_2_O_7_ at temperatures in the range of 500–600 °C, which further converts into the more stable β-Ca_2_P_2_O_7_ and α-Ca_2_P_2_O_7_ forms at ~700–800 °C and 1000–1200 °C, respectively, in air ambient [57,58,59] and at slightly more elevated temperatures in nitrogen [50]. Concurrently, up to 1200 °C, HA was found to gradually decompose into β-TCP or a mixture of β-TCP and α-TCP [19,60]. The high temperature de-hydroxylation of HA (with the intermediate formation of oxyapatite, regardless of the sintering ambient [51,52]) is known to be the main promoter of its conversion to β-TCP [53,61,62]. Thereby, the presence of the minor oxyapatite phase for the air sintered specimens (Figure 1a,b) could be interpreted as an indication that the de-hydroxylation process of HA already began, suggesting that its decomposition can be expected at temperatures in excess of 1200 °C. In addition to de-hydroxylation, the interaction of HA with the Ca_2_P_2_O_7_ counterpart when the sintering temperature is increased is considered to be a second contributor to the decomposition into β-TCP [62]. However, the origin of the TCP phases cannot be solely ascribed to HA as the hypothesis of thermal decomposition of DCPD into both Ca_2_P_2_O_7_ and TCP phases was also advanced [50]. The appearance of the α-TCP phase is now well-established to originate from the displacive transformation of β-TCP at temperatures of 1100–1200 °C or higher [2,28,29,53,63]. The hexaphosphate phase, although adventitiously formed in reduced amounts, is also a subsidiary effect of the DCPD sintering, yet was previously signaled in air ambient [18] and not in nitrogen.
(ii). The dissimilarity in crystalline phase composition of the samples sintered in air ambient (Figure 1a,b), with respect to those processed in nitrogen (regardless of their compactness) (Figure 1c,d), is undoubtedly enabled by the sintering environment. This was to be expected since scientific literature evidence advocates that a less-reactive ambient alone, such as nitrogen, favors an accentuated de-hydroxylation of the HA and, hence, a diminished structural stability at high temperatures (i.e., 1200 °C), shifting the equilibrium to both β- and α-TCP phases [12,52].
(iii). The presence of Gr can be viewed as a supplemental adjuvant (until its complete consumption) for the β-TCP → α-TCP conversion under nitrogen ambient. This is markedly evident for the CP-type specimens (Figure 2a), composed of closely packed ceramic particles admixed with Gr. This phenomenon can be ascribed to the generation of a higher thermal gradient (as Gr has a significantly higher thermal conductivity (3000 W/m·K) with respect to CaPs (1.1–1.25 W/m·K) [10,25,64]), which in turn, can foster an accelerated phase transformation [25,52].

Hence, the transformation of the HA/DCPD material into β-TCP or a mix of β-TCP/α-TCP/α-Ca_2_P_2_O_7_ is suggested to be coordinated by the source materials features and its heat decomposition dynamics, the sintering ambient, and the presence/absence of Gr.

### 3.2. FTIR Spectroscopy Analysis

**Figure 3:**
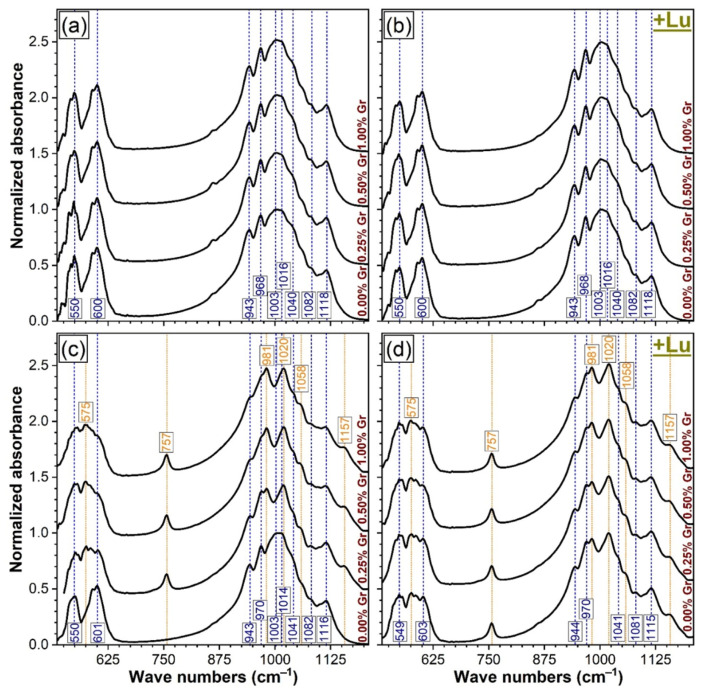
The comparative FTIR-ATR spectra of the (**a**,**c**) compact and (**b**,**d**) porous samples thermally-treated in (**a**,**b**) air and (**c**,**d**) nitrogen ambient at 1200 °C/8 h.

**Figure 4:**
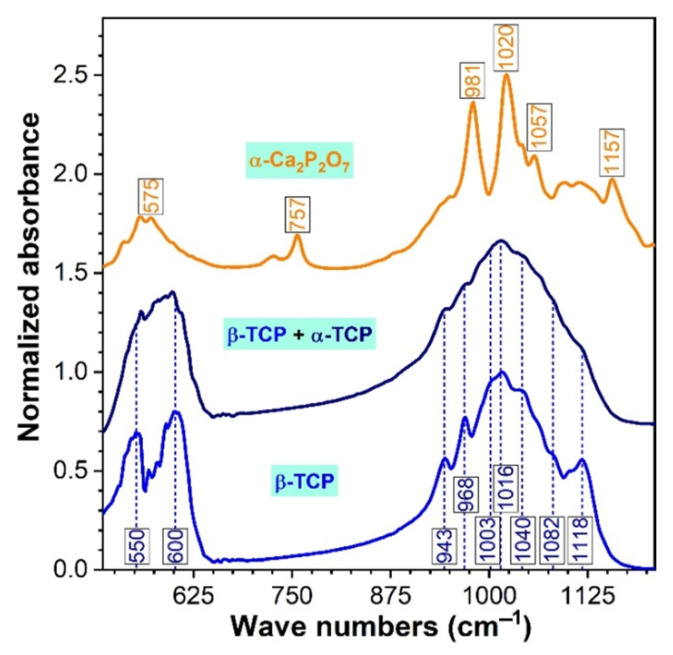
The FTIR-ATR spectra of the control samples: pure β-TCP (Sigma-Aldrich), β-TCP + α-TCP blend (obtained by heat-treating the Sigma-Aldrich β-TCP powder at 1400 °C/4 h in air) and α-Ca_2_P_2_O_7_ (synthesized by co-precipitation, see ref. [16]).

The FTIR-ATR spectra of all CP and PP specimens, synthesized under air and nitrogen ambient, are displayed comparatively in Figure 3. The FTIR-ATR spectroscopy data were found in excellent agreement with the earlier-presented XRD data (Figure 1 and Figure 2). Specifically, it was noticed that the band positions, shapes, and amplitudes of all air heat-treated samples (Figure 3a,b) closely mirrored the spectral envelope obtained in the case of the pure β-TCP commercial powder (Figure 4). All the orthophosphate characteristic vibrational bands of a β-TCP compound were featured, i.e., ν_4_ asymmetric bending (~550 and 600–601 cm^−1^), ν_1_ symmetric stretching (~943 cm^−1^), and the ν_3_ triply degenerated asymmetric stretching (~968–970, 1003, 1014–1016, 1040–1041, 1082, and 1118 cm^−1^) [16,63]. In the case of the air sintered specimens only, the characteristic IR bands of β-TCP were evidenced (Figure 3a,b), thus, confirming the XRD phase identification (Figure 1a,b).

The decomposition of β-TCP phase into the α-TCP and the supplemental presence of α-Ca_2_P_2_O_7_ (Figure 3c,d), recorded in the case of nitrogen thermally-treated samples (with the exception of the compact 0.00 wt.% Gr one), was exposed as well by occurrence of two concomitant events: (i) the β-TCP bands became less conspicuous (as result of the juxtaposition of the IR absorption maxima of both β-TCP and α-TCP, leading to convoluted bands, as evidenced by both our experiments in the case of the β-TCP + α-TCP blend (Figure 4) and Carrodegues et al. [63] and (ii) the emergence of well-defining protruding bands characteristic to the vibrational modes of a pyrophosphate phase (Figure 4): asymmetric bending (~575 cm^−1^), symmetric stretching of P–O bridges (~757 cm^−1^), asymmetric stretching of P–O bridges (~981 cm^−1^), symmetric stretching mode of terminal P–O (~1020 cm^−1^), or asymmetric stretching mode of terminal P–O (~1057–1058 cm^−1^ and ~1157 cm^−1^) [16,59], superimposed over the dominant TCP spectral envelope.

In good correspondence to the XRD results, the intensity reduction and convolution of the well-defined IR band at ~943–944 cm^−1^ testifies for the emergence of the α-TCP phase (similarly to XRD, the incremental reduction of this band intensity for the CP specimens sintered under nitrogen ambient indicates the gradual transformation of β-TCP into α-TCP). The virtually constant intensity of the prominent IR band of α-Ca_2_P_2_O_7_ (at ~757 cm^−1^), with respect to the amplitude of the FTIR peaks of TCP, suggests that the CPP content does not decidedly modify with the Gr and/or Lu addition.

### 3.3. Morpho-Compositional SEM/EDS Evaluation

**Figure 5:**
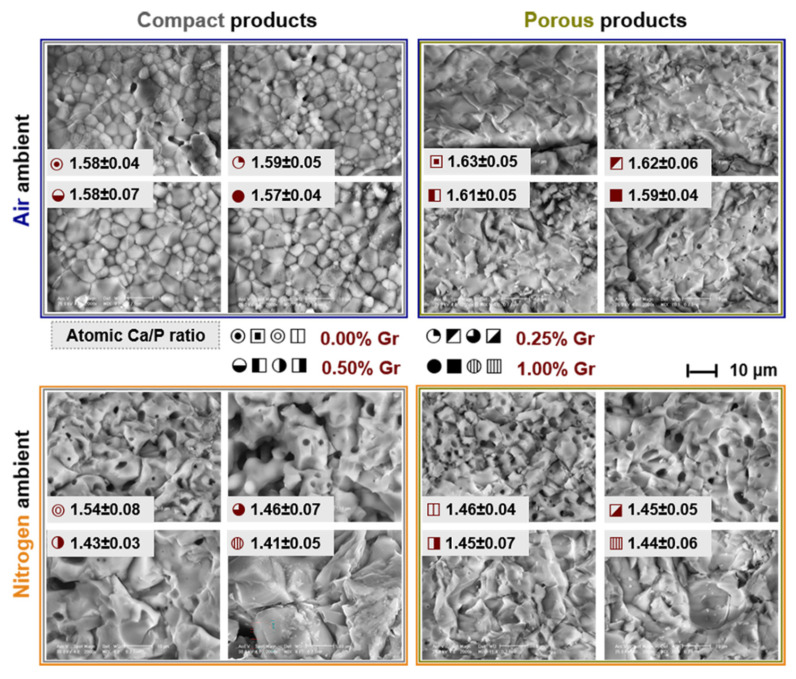
The morpho-compositional evolution of the compact and porous samples thermally-treated in air and nitrogen ambient at 1200 °C/8 h.

The morphological evaluation results performed on fractographic surfaces of air and nitrogen thermally-treated samples are comparatively summarized in Figure 5.

For all type of samples, the fractography analyses revealed a brittle inter-crystalline crack propagation, with few areas of intra-crystalline fracturing recorded only for samples with ≥0.50 wt.% Gr. The ceramics consisted of prominent polyhedral faceted grains with well-defined grain boundaries, regardless of the sintering ambient. Additionally, the micrographs evidenced improved interlocked grains for samples sintered in air and confirmed the elimination of the Gr material from all products.

In air ambient, the grain sizes and the formation of the few residual pores slightly increased for the samples with the highest Gr amount. In addition, the sintering process in nitrogen ambient led to an accentuated uniform microporous structure, which gradually formed with the addition of the Gr agent. The largest pores, formed at the grain boundaries or on the facets of the enlarged grains, were spherically shaped. This evolution can be attributed to the consolidation process of the reinforced ceramic matrix, during which, under the applied force, the graphene nanoplatelets are either bent, distributed alongside ceramic particle edges, or embedded between them [47], owing to the favorable interaction between the two materials [65]. The ability of Gr to generate variable sized pores under high temperature was previously attested [44,45]. Studies revealed that, during the thermal treatment, Gr may prevent the successful coalescence of all ceramic particles, leading to an open porosity and a less-densified microstructure. However, the occurrence of a secondary porosity with bone-like pore sizes was shown as beneficial for cell adhesion and proliferation, fluid transportation, and/or nerve accommodation [8].

The densification phenomenon, translated into progressive grain growth, is favored by the nitrogen ambient and by the increase of the Gr content, as previously advertised for other inert or reductive sintering environments [66]. However, one must take into account that, under these sintering conditions, the ceramic matrix also suffers severe structural transformations, as highlighted in Figure 1, Figure 2, Figure 3 and Figure 4, which further contribute to the recorded morphological evolution. The development of extensive grains coincides with the emergence and gradual increment of the α-TCP phase, which is remarkably pronounced in the case of compact products (Figure 2). Previous results depicted that the thermal expansion coefficient and the existent β-TCP volume are directly proportional, leading to a detrimental effect upon densification at higher temperatures due to its further transformation into α-TCP [2,28,29,67]. The Ca_2_P_2_O_7_ phases were also found to prevent a proper densification of the sintered samples [2].

Moreover, in the case of porous products processed in both types of sintering environment (air and nitrogen), the gaseous products released during the combustion (volatile elimination and/or CO_2_ adsorption) of Lu fibers, act as major incentives for an increased porosity of the ceramic matrix, along with the creation of internal interconnected channels (see Figure 7) [4,40,48].

Another stringent factor linked to the future performance of the sintered products is the atomic Ca/P ratio. The Ca/P values, determined based on the EDS quantitative analyses, were in agreement with the structural events evidenced by XRD and FTIR spectroscopy investigations (Figure 1, Figure 2, Figure 3 and Figure 4). For all products, in both air and nitrogen ambient, with/without Lu fibers (Figure 5), descending trend-lines were outlined with the increase of the Gr amount; the lower values calculated for samples treated in nitrogen could be directly correlated with the obtained mixed compositions [2,39,45,68,69].

### 3.4. Dimensional Shrinkage and Mass Loss Determination

**Figure 6:**
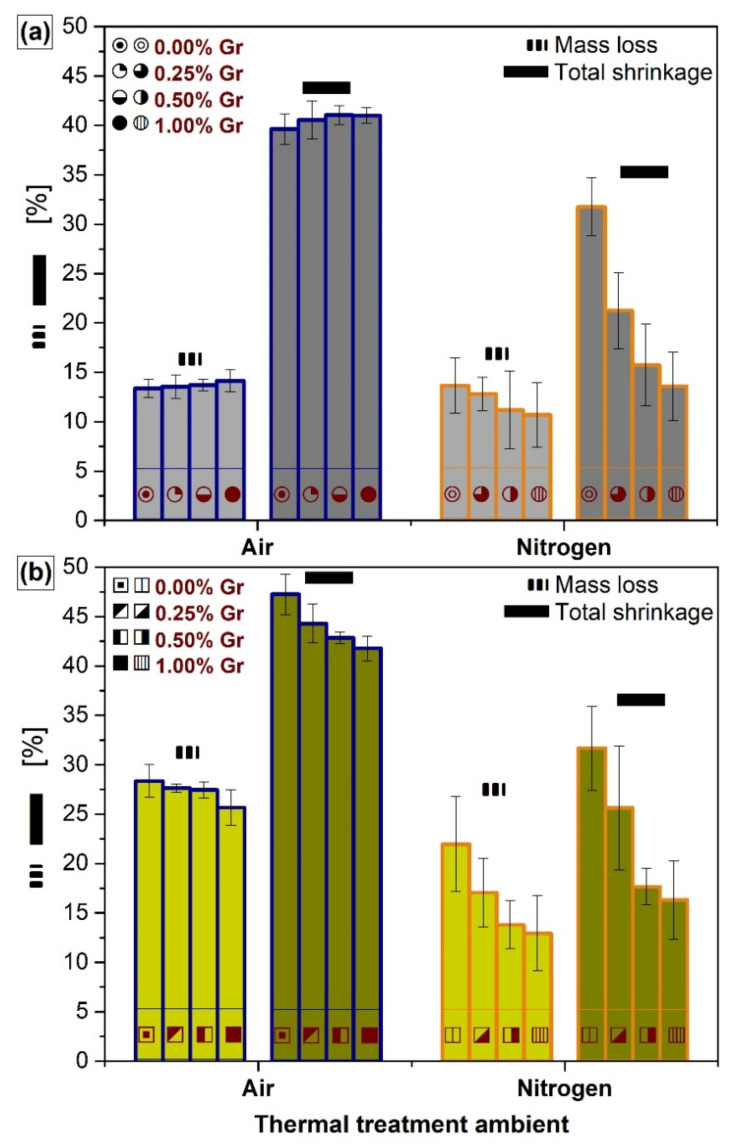
The comparative mass loss and total shrinkage of the (**a**) compact and (**b**) porous samples thermally-treated in air and nitrogen ambient at 1200 °C/8 h.

The evolution of the mass loss and total shrinkage of the compact and porous products, obtained by sintering in air and nitrogen ambient, is comparatively displayed in Figure 6a,b and Tables S1 and S2 (Supplementary Materials). The formulas for the determination of the aforementioned parameters are described in refs. [33,60].

The sintering process is essential for the shape consolidation of the desired bioceramic products and operates by the elimination of all organic, volatile, or humid agents [32,70]. Taking into account the two modulated parameters (i.e., Gr amount and sintering ambient), the obtained results are in good agreement with the structural and morphological evolutions presented in Figure 1, Figure 2, Figure 3, Figure 4 and Figure 5.

All samples sintered under air ambient elicited significant mass loss and total shrinkage. Opposite trends were observed for the compact and porous samples once the Gr amount is increased, as suggested by the higher micro-porosity recorded for the PP samples (as evidenced in Figure 5). The negative influence of a higher micro-porosity upon the shrinkage degree in the case of PP was previously discussed [6]. Apart from this, even though the actual Gr amount for the PP samples is lower due to the incorporation of Lu fibers (as detailed in the samples preparation section), the results suggested that the thermal removal of Lu is directly influencing the final dimensions of the products. Hence, greater values for the linear mass loss and total shrinkage were plotted, as compared to the CP samples, with augmented factors of 2.12–1.81 and 1.19–1.02, respectively, decreasing with the Gr amount.

Nonetheless, along with the contrasting structural (Figure 1 and Figure 3) and morphological (Figure 5) changes induced by the nitrogen ambient, a uniform shrinkage tendency, decreasing with the incremented addition of Gr, was also detected. The effect of complete transformation of HA and DCPD into β-TCP, and its further partial transition into α-TCP, with the increase of the admixed Gr, accounts for the sudden shrinkage arrest [29,49,67]. Apart from the Gr influence, the appearance of the Ca_2_P_2_O_7_ phase is also linked with a fast and abnormal grain growth and can additionally assist the expansion of the sample volume [2]. The same tendency trajectory as in the air ambient can be depicted for the PP samples, where the complete removal of Lu fibers leads to an accentuated dimensional reduction by 1.61–1.22 and 1.21–1.0 for the mass loss and total shrinkage, as compared to the CP samples.

Overall, the results revealed that the higher the admixed graphene amount is, the higher the inhibition behavior towards shrinkage is. This is even more pronounced in nitrogen ambient, where both mass loss and total shrinkage recorded diminished values in the case of both CP and PP samples. Generally, the porosity and structural modifications need to be compromised in certain extents so as to obtain controlled shrinkage degrees and optimal architectural and mechanical features [32].

### 3.5. Nano-CT Reconstruction Analysis

**Figure 7:**
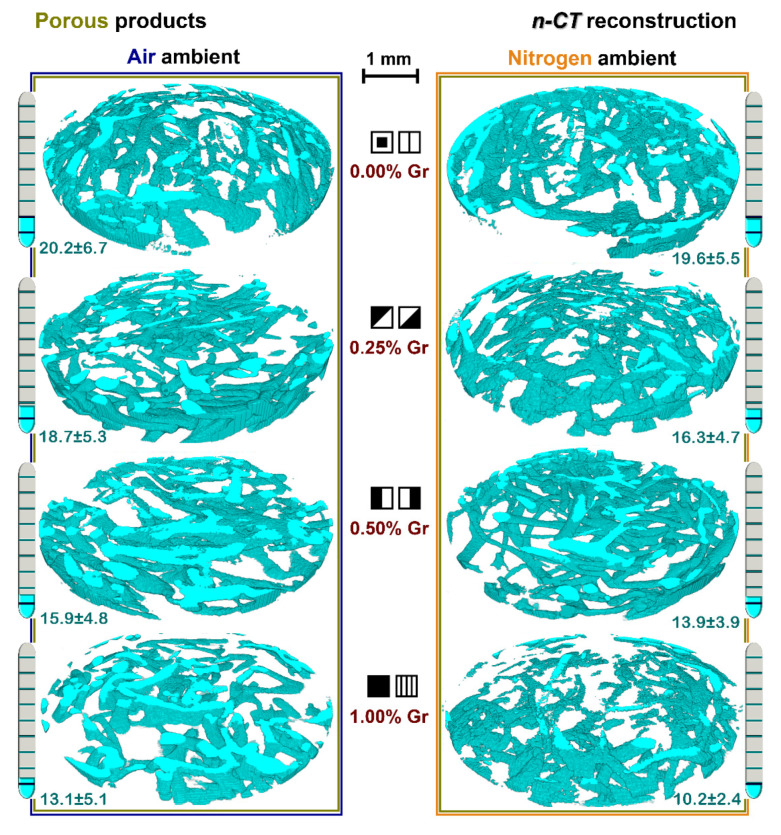
Nano-CT 3D reconstruction of the porous samples thermally-treated in air and nitrogen ambient at 1200 °C/8 h. The porosity variation of all samples is provided as percentage bars on the sides of the figure, along with the calculated values (*n* = 5, mean ± SD).

The three-dimensional (3D) nano-CT reconstructions of the PP samples sintered in air or nitrogen ambient at each Gr amount are presented in Figure 7. This non-destructive imaging technique allows for the visualization of complex surfaces and internal architectures in terms of spatial heterogeneity of pores and channels throughout the entire product, as compared to the surface-limitation of the frequently used complementary SEM method [71,72,73]. In this regard, the pore distribution, size, and geometry and, most importantly, the interconnectivity and tortuosity degree, frame the usefulness of this processing approach for various materials (ceramics included) [74].

Herein, the set-up of the software parameters offered the possibility to display the results function of the area of interest, namely, the structure of the generated channels (colored in blue). The blank areas of the samples, virtually seen as voids, consist, in fact, of the ceramic matrix of the bio-products enclosing the channels. The nano-CT results confirmed, in the case of all samples, that the degradation of the sacrificial porogen template (Lu fibers) was complete, and that complex arrays of pores and remarkable hollow microchannels were successfully induced at high temperatures without any residual chars/combustion products, regardless of the sintering ambient. Additionally, the micro-porous aspect, induced by the release of volatiles and gaseous products from the thermally-treated fibers [40]), which further impregnate on the internal surface of the channels [4,10], is confirmed also from the outer view of the channel walls.

Given the multidirectional arrangement and specific anastomosis of the Lu fibers, the developed channels preserved an arbitrary and tortuous surface–volume distribution in the entire ceramic mass at all incorporated Gr amounts in air ambient. However, only at low Gr amounts (0.25–0.50 wt.%) did the channel diameters have comparable sizes to those of the thermally-treated Lu fibers (around 200–350 μm), previously assessed and reported in Ref. [4], and predominantly round shapes. The increase of the Gr amount up to 1.00 wt.% translates into the generation of flattened geometries (as recently remarked also in the case of the developed large surface pores that replaced the fibers [10]), which further led to few cleaved channel lengths and an overall reduced number of formed channels. As one can also note, the samples sintered in nitrogen ambient gradually shifted from uniform architectures to reduced, deformed, and dot-like sectioned channels from the incipient up to the maximum Gr amount. These findings are also supported by the calculated total porosity (Figure 7) based on the segmented images, which exposed similar downward trends as the Gr amount increased for both air and nitrogen treated samples; an accentuated decreased porosity was clearly noticed for the latter ones (from ~19 to ~10% as compared to ~20 to ~13%). The observed phenomena can be yet another effect of the above-described structural and morphological evolutions of the samples. Previous studies regarded the Lu fibers as highly resistant to applied mechanical forces [36] and prone to preserve the original tubular structure when treated in nitrogen ambient [75]. However, the thermal expansion of the samples due to substantial grain growth and pore formation in the ceramic matrix once the Gr amount was increased at 1.00 wt.% (Figure 5) impacted the shrinkage effect to lower extents (as indicated in Figure 6), yet impeded the complete transition and attainment of proper pores and channels. It led to a preferential congestion of the channels towards the product surfaces.

Such behaviors implicitly operate against the optimum continuity and interconnectivity of the internal channel arrangement and of the total bulk porosity, desired to closely mimic the vascular-like network of the human bone and foster an optimum fluid flow, and the natural porosity level (e.g., >40% for the cancellous bone) [32]. Nevertheless, different type of pores and channel geometries proved to be compatible with an adequate angiogenesis and bone formation rate required for orthopedic reconstruction applications [31,76].

Therefore, the practicality of Lu fiber use as porogen template is restricted by the concomitant addition of the Gr agent (as mechanical reinforcement agent) up to maximum 0.50 wt.% when the sintering program is conducted in nitrogen ambient. Other than that, the proposed 3D geometries comply with the tortuosity and interconnectivity degree, the pore and channel sizes (around 100–500 μm) [5,8,71], and can stand as precursor arrays for future osteogenesis when implemented in various bone healing applications. Since the mechanical features are also of great importance for favorable in vivo performance of the scaffolds, a compromise can be attained with respect to the porosity degree [32], by modulating the amounts of Lu fibers and/or Gr agents.

### 3.6. Contact Angle Measurements

**Figure 8:**
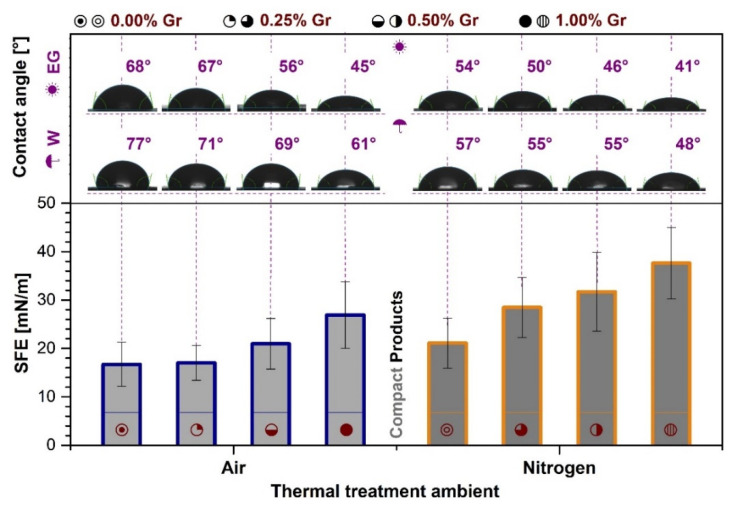
Contact angle measurements—performed with two wetting agents: water (W) and ethylene glycol (EG) and surface free energy (SFE) results for the compact samples thermally-treated in air and nitrogen ambient at 1200 °C/8 h.

The wettability and surface free energy responses could only be obtained for the CP samples (Figure 8). The surface properties (i.e., topography, wettability, surface energy) of an implantable product determine its prospected capacity to be integrated into the host tissue and return a positive biological response [76,77]. Thereby, due to the high surface–volume porosity and interconnected channels distributed in the ceramic matrix, as evidenced by the nano-CT scans (Figure 7) and previous results [39], the absorption of both testing fluids developed instantly after the droplet deposition on the surface of the PP products. This fast evolution impeded the acquisition of accurate contact angle data but, at the same time, attested that their morphology is favorable for fluid absorption and cell colonization.

Regarding the wettability of CP samples, the W contact values ranged between 77°→61° and 57°→48°, while the EG ones displayed slightly decreased trend lines from 68° to 45° and 54° to 41°, with the increment of the admixed Gr amount, for samples treated in air and nitrogen ambient, respectively. Both sets of experimental data revealed the hydrophilic character of all samples (contact angle values <90°). As expected, the evolution of the samples towards higher porosity degrees (a key factor for contact angle evaluation [55]) can be further linked to the enhanced wetting capacity observed for samples with higher Gr amounts (0.50 and 1.00 wt.%), regardless of the sintering ambient. For both types of sintering ambient, once the 0.25 wt.% Gr limit was exceeded, the contact angle values for the dispersive agent dropped abruptly, while the polar component was similarly influenced above 0.50 wt.% Gr. However, the overall behavior was considerably accentuated for samples treated under nitrogen ambient.

The corresponding SFE values, also displayed in Figure 8, increased with the Gr amount, the nitrogen ambient seemingly having a more pronounced influence. A higher value of the surface free energy is an indicator of an increased hydrophilicity. In this regard, the 50–80° range was suggested to be optimal for a proper wettability and cell survival [78,79,80,81]. This translates into an improved future protein adsorption and cell adhesion and proliferation on a given sample surface, leading to an augmented biological response [82].

### 3.7. Mechanical Properties Evaluation

**Figure 9:**
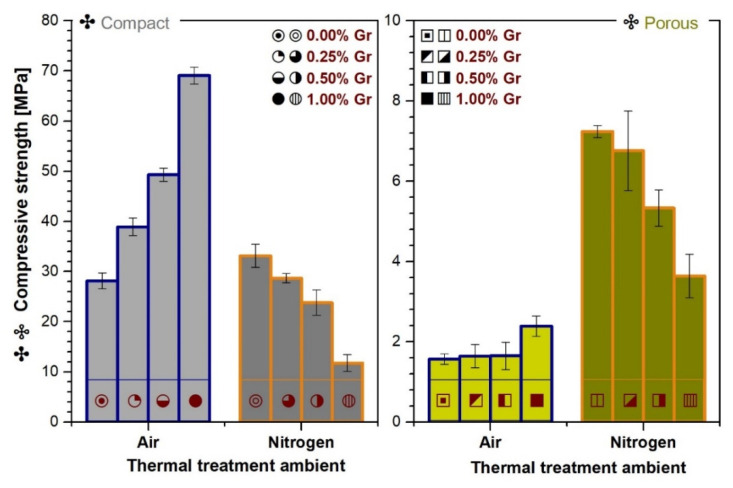
Comparative compressive strength results of the compact and porous samples thermally-treated in air and nitrogen ambient at 1200 °C/8 h.

The mechanical behavior of CP and PP samples treated in air and nitrogen ambient was evaluated by uniaxial compression testing, and the results are comparatively presented in Figure 9.

At first glance, one can easily note that the mechanical features evolve preferentially for the CP and PP samples, function of the sintering ambient. The variation of the compressive strength for both sample types, is also linked to the absence/presence of Gr and implicitly to the morphological and structural aspects depicted in Figure 1, Figure 2, Figure 3, Figure 4 and Figure 5.

The air sintering process, combined with gradually increased Gr amounts, promoted a linear ascending tendency of the compressive strength values for both CP and PP products. In agreement with our previous preliminary study [10], at the maximum Gr amount (1.00 wt.%) the compressive strength more than doubles for the CP samples and increases by 1.3 for the PP ones, as compared to samples consisting of only the ceramic matrix with/without Lu fibers. The reinforcing capacity of the Gr agent usually translates into an overall improvement of the fracture resistance and compressive strength. Still, the addition of Gr materials in higher doses was sometimes found to be detrimental [41,44,45,47].

In the case of nitrogen sintered specimens, the compressive strength values were in complete opposition. For CP samples, the presence of Gr slightly increased the mechanical resistance only for the minimum admixed amount (0.25 wt.%), after which, up to 1.00 wt.%, the performance dropped abruptly to almost half the value corresponding to the air sintered ceramic matrix (0.00 wt.% Gr). In contrast, a favorable evolution was depicted for the PP samples, for all Gr amounts, suggesting that the Lu fibers are also at play for the mechanical reinforcement mechanism. It was previously implied that the release of the gaseous products, as a result of fiber thermal degradation, improves the mechanical plasticity and the stress and load distribution throughout the samples, in case of crack formation [10]. A fully detailed description of the combustion process for Lu fibers in both air and nitrogen ambient is provided in ref. [4].

Although significantly higher when compared to the air ambient, the values recorded in nitrogen ambient for the PP samples still decreased with the increase of the Gr agent, similarly to the CP ones. The backsliding tendencies of the compressive strength values in nitrogen ambient can be attributed to the detected shifts in the phase composition and morphology. The correlated appearance of extensive grain growth (Figure 5) and gradual transition of β-TCP into α-TCP (for CP samples produced under nitrogen ambient) with the increase of the Gr amount sustain the fracture initiation and intergranular crack propagation similar to cleavage planes, leading to more fragile products and jeopardizing the mechanical features, as previously reported [2,8,28,45,83]. Another prospected factor with dramatic effects in this regard is constituted by the porosity degree—the higher the porosity degree or pore sizes, the lower the mechanical resistance [12,14,25,44]. This explains the radical drop of values at the highest Gr amount in the case of CP samples, based on the open porosity and abnormal pore sizes found in the matrix. However, as the nano-CT scans revealed in the case of the PP samples obtained under nitrogen ambient, the overall porosity degree and channel dimensions varied inversely with the Gr amount. Hence, the reduction effect on the interconnected channels exceeded that of the surface morphology of the ceramic matrix, leading to over three times greater compressive strength values (for 0.25 wt.% Gr).

Nevertheless, given the biological application-target for both type of products, the nano- and micro-scaled pores and porosity are necessary for an excellent osteoconductivity as they could facilitate the proper osteoblast cell growth on or within the bio-products [5,25]. On one hand, for an adequate mechanical strength, the developed structure requires solid grounds with controlled morphological (grain and pore sizes, porosity) and compositional features, since all constitute important factors, while cell colonization is mostly facilitated by interconnected pores and channels. Also, porosity contributes to the fluent exchange of nutrients, blood vessel formations, and metabolic system function at bone level. Therefore, the porosity and mechanical integrity should be balanced accordingly, as they are defining criteria for quality bone graft substitutes [14].

Considering all the above-mentioned aspects and the requirement for a greater resemblance to the natural bone behavior, optimal mechanical results, close to those of the cortical and cancellous bone [8,27,84], were obtained for all samples, treated in both air and nitrogen ambient, except for the compact samples with the maximum Gr amount. In the latter case, the compressive strength performance is rather more applicable for the cancellous bone reconstruction than the cortical one.

## 4. Conclusions

The comparative effects of the sintering ambient (air or nitrogen) on the structure, morphology, surface energy, and mechanical performance of compact and porous bio-products, fabricated based on a one-stage binding free preparation route by the addition of graphene nanoplatelets and *Luffa* fibers as mechanical reinforcement and porogen agents, respectively, were explored for the first time.

Under an air sintering environment, products consisting of a β-TCP dominant crystalline phase were produced, irrespective of the graphene content or *Luffa* fiber incorporation. The nitrogen ambient favored an accentuated decomposition of the original HA/DCPD ceramic matrix, leading to variable proportions of the β-TCP, α-TCP, and Ca_2_P_2_O_7_ phases for both compact and porous type products. Significant morphological changes were induced in terms of extended grain growth and pore formation in the ceramic matrix. The mechanical features of the sintered products fit very well with the recorded structural and morphological trends.

A series of technological guidelines can be derived. For compact products: (i) augmented compressive strengths are attained in air ambient with the increase of the Gr agent; (ii) if the nitrogen ambient is desired, then the Gr amount should be limited to maximum 0.25 wt.%. Conversely, for porous products: (i) neither of the sintering environments interfere with the ability of *Luffa* fibers to generate bone-like architectures with interconnected channels of variable sizes; (ii) structural shifts and mechanical improvements can be attained under nitrogen ambient; and (iii) the concomitant association of *Luffa* fibers, Gr nanoplatelets, and nitrogen ambient acts as adjuvant for the fabrication of value-added structures, possibly for an extended range of healthcare applications.

In the long term, depending on the nature of the orthopedic application and its required compositional, morphological, architectural, and mechanical features, the *Luffa* fibers/Gr material ratio can be accordingly modulated so as to achieve beneficial outcomes after sintering in any of the two investigated sintering environments.

## Data Availability

All data generated or analyzed during this study are included in this published article. The raw data can be made available from the authors upon reasonable request.

## Supplementary Materials

The following are available online at https://www.mdpi.com/article/10.3390/ma14092198/s1, Figure S1: The step experimental procedure for the incorporation of the two agents (Graphene and Luffa fibres) into the ceramic matrix and the development of both compact and porous samples. Visual assessment of the samples prior and post sintering in both ambients (air and nitrogen); Table S1: Experimental measurements of compact pellets sintered in air and nitrogen ambient; Table S2: Experimental measurements of porous pellets sintered in air and nitrogen ambient.
