# Supplementary material for: Fiber-Templated 3D Calcium-Phosphate Scaffolds for Biomedical Applications: The Role of the Thermal Treatment Ambient on Physico-Chemical Properties
Source: Materials (Basel). 2021 Apr 25;14(9):2198. doi: 10.3390/ma14092198 (PMC8123353; doi:10.3390/ma14092198)
Supplement: Supplementary file 1 [file materials-14-02198-s001.zip › materials-1191994-supplementary.pdf]

materials

Supplementary Materials

Fiber-Templated 3D Calcium-Phosphate Scaffolds for Biomedical Applications: The Role of the Thermal Treatment Ambient on Physico-Chemical Properties

Aura-Cătălina Mocanu [1], Florin Miculescu [1,*], George E. Stan [2], Andreea-Mădălina Pandele [3,4], Mihai Alin Pop [5], Robert Cătălin Ciocoiu [1], Ștefan Ioan Voicu [3] and Lucian-Toma Ciocan [6]

[1] Department of Metallic Materials Science, Physical Metallurgy, University Politehnica of Bucharest, 313 Splaiul Independentei, J Building, RO-060042 Bucharest, Romania; mcn_aura@hotmail.com (A.-C.M.); ciocoiurobert@gmail.com (R.C.C.)
[2] National Institute of Materials Physics, 405A Atomistilor Street, RO-077125 Măgurele, Romania; george_stan@infim.ro
[3] Department of Analytical Chemistry and Environmental Engineering, University Politehnica of Bucharest, 1–7 Gh. Polizu, RO-011061 Bucharest, Romania; pandele.m.a@gmail.com (A.-M.P.); svoicu@gmail.com (Ș.I.V.)
[4] Advanced Polymer Materials Group, University Politehnica of Bucharest, 1–7 Gh. Polizu, RO-011061 Bucharest, Romania;
[5] Department of Materials Science, Faculty of Materials Science and Engineering, ICDT, University Transilvania of Brasov, 10 Institutului, RO-500484 Brasov, Romania; mihai.pop@unitbv.ro
[6] Prosthetics Technology and Dental Materials Department, "Carol Davila" University of Medicine and Pharmacy, 37 Dionisie Lupu Street, RO-020022 Bucharest, Romania; tciocan@yahoo.com
* Correspondence: florin.miculescu@upb.ro; Tel.: +40–21–316-95-63

Citation: Mocanu, A.-C.; Miculescu, F.; Stan, G.E.; Pandele, A.-M.; Pop, M.A.; Ciocoiu, R.C.; Voicu,S, .I.; Ciocan, L.-T. Fiber-Templated 3D Calcium-Phosphate Scaffolds for Biomedical Applications: The Role of the Thermal Treatment Ambient on Physico-Chemical Properties. *Materials* **2021**, *14*, 2198. https://doi.org/10.3390/ma14092198

Academic Editor: Andrew Ruys

Received: 5 April 2021
Accepted: 23 April 2021
Published: 25 April 2021

Publisher's Note: MDPI stays neutral with regard to jurisdictional claims in published maps and institutional affiliations.

Copyright: © 2021 by the authors. Licensee MDPI, Basel, Switzerland. This article is an open access article distributed under the terms and conditions of the Creative Commons Attribution (CC BY) license (http://creativecommons.org/licenses/by/4.0/).

FIGURES

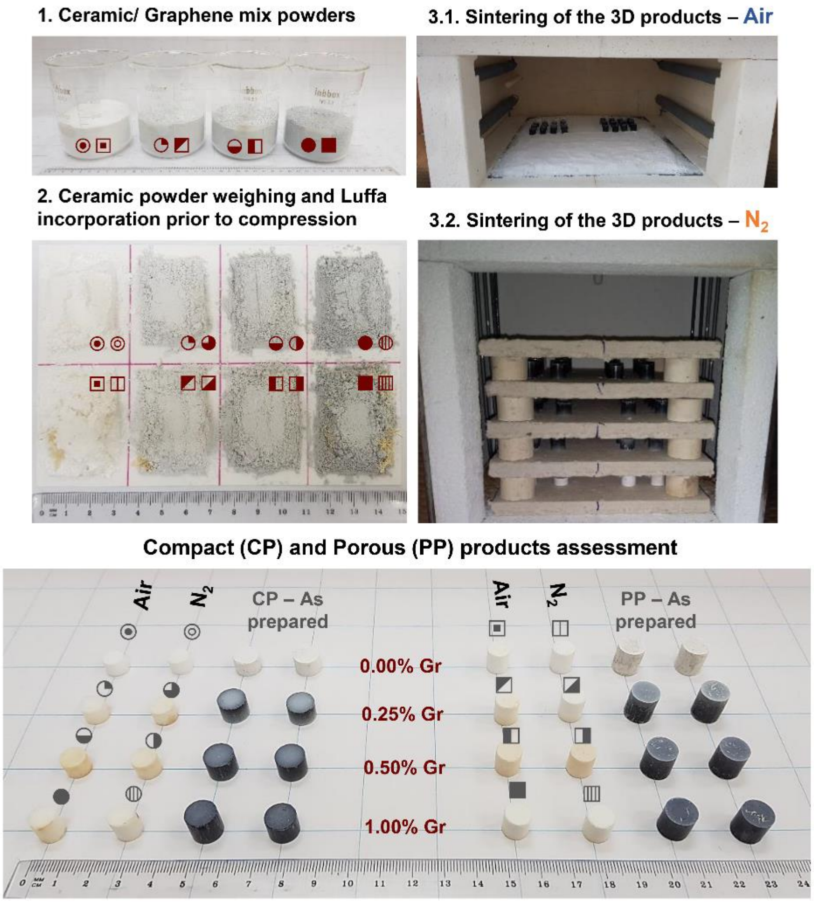

Figure S1. The 3-step experimental procedure for the incorporation of the two agents (Graphene and *Luffa* fibres) into the ceramic matrix and the development of both compact and porous samples. Visual assessment of the samples prior and post sintering in both ambients (air and nitrogen).

TABLES

Table S1. Experimental measurements of compact pellets sintered in air and nitrogen ambient.

	Sample	Mass [g]		Height [mm]		Diameter [mm]		Volume [mm³]	
		m_i	m_f	h_i	h_f	d_i	d_f	v_i	v_f
Air	**0.00% Gr**	1.288 ±0.101	1.116 ±0.086	8.180 ±0.708	6.878 ±0.545	10.315 ±0.079	8.670 ±0.014	683.221 ±55.282	405.825 ±30.734
	**0.25% Gr**	1.516 ±0.175	1.311 ±0.151	9.168 ±1.420	7.738 ±1.102	10.308 ±0.043	8.710 ±0.078	764.588 ±118.178	460.794 ±61.540
	**0.50% Gr**	1.566 ±0.094	1.352 ±0.081	9.583 ±0.844	7.945 ±0.583	10.335 ±0.019	8.710 ±0.018	803.470 ±68.121	473.151 ±33.314
	**1.00% Gr**	2.006 ±0.141	1.722 ±0.121	12.288 ±1.345	10.355 ±1.030	10.253 ±0.067	8.575 ±0.070	1013.894 ±99.287	597.707 ±50.549
Nitrogen	**0.00% Gr**	1.663 ±0.322	1.477 ±0.290	9.665 ±1.913	8.539 ±1.677	10.334 ±0.025	9.077 ±0.162	810.230 ±161.715	552.283 ±110.835
	**0.25% Gr**	1.821 ±0.086	1.627 ±0.108	10.196 ±0.608	9.590 ±0.689	10.331 ±0.010	9.448 ±0.257	854.248 ±51.639	671.998 ±54.070
	**0.50% Gr**	1.801 ±0.067	1.553 ±0.095	10.189 ±0.553	9.930 ±0.623	10.330 ±0.050	9.705 ±0.298	853.497 ±46.008	734.193 ±80.512
	**1.00% Gr**	1.990	1.735	10.814	10.649	10.334	9.559	906.552	763.842

±0.132	±0.127	±0.790	±0.836	±0.051	±0.140	±66.636	±51.337

Table S2. Experimental measurements of porous pellets sintered in air and nitrogen ambient.

	Sample	Mass [g]		Height [mm]		Diameter [mm]		Volume [mm³]	
		m_i	m_f	h_i	h_f	d_i	d_f	v_i	v_f
Air	0.00% Gr	1.870	1.341	12.650	8.905	10.298	8.905	1052.989	554.334
		±0.092	±0.097	±0.568	±0.078	±0.075	±0.078	±46.237	±14.623
	0.25% Gr	1.931	1.399	12.663	10.993	10.320	8.910	1058.641	685.049
		±0.175	±0.151	±1.420	±1.102	±0.043	±0.078	±118.178	±61.540
	0.50% Gr	1.853	1.378	11.753	10.215	10.283	8.975	975.433	645.917
		±0.044	±0.041	±0.355	±0.326	±0.039	±0.034	±31.847	±17.367
	1.00% Gr	1.784	1.294	11.303	9.795	10.348	8.973	949.981	619.015
		±0.044	±0.017	±0.436	±0.410	±0.055	±0.053	±33.448	±29.337
Nitrogen	0.00% Gr	1.736	1.510	10.788	9.554	10.321	9.252	902.099	641.987
		±0.350	±0.297	±2.388	±2.089	±0.026	±0.171	±202.183	±132.129
	0.25% Gr	1.658	1.429	9.487	8.721	10.325	9.475	793.924	614.588
		±0.090	±0.119	±0.537	±0.844	±0.011	±0.259	±45.955	±58.424
	0.50% Gr	1.741	1.445	10.170	9.765	10.331	9.683	852.070	718.723
		±0.105	±0.171	±0.525	±0.538	±0.030	±0.077	±44.125	±42.338
	1.00% Gr	1.811	1.414	10.056	9.893	10.327	9.635	841.867	720.943
		±0.090	±0.197	±0.532	±0.609	±0.050	±0.049	±44.554	±46.977
